# Comparative Analysis of Methicillin-Susceptible *Staphylococcus aureus* and Streptococcal Native Joint Septic Arthritis: Clinical Characteristics and Outcomes

**DOI:** 10.3390/antibiotics15070714

**Published:** 2026-07-22

**Authors:** Jungok Kim, Eun-Jeong Joo, Ki-Ho Park, Bomi Kim, Mi Suk Lee

**Affiliations:** 1 Division of Infectious Diseases, Department of Medicine, Chungnam National University School of Medicine, Dae-Jeon 35015, Republic of Korea; kjo@cnuh.co.kr; 2 Division of Infectious Diseases, Department of Internal Medicine, Kangbuk Samsung Hospital, Sungkyunkwan University School of Medicine, Seoul 03181, Republic of Korea; bomi.id.kim@samsung.com; 3 Department of Infectious Diseases, Kyung Hee University College of Medicine, Kyung Hee University Hospital, Seoul 02447, Republic of Korea; mslee7@khu.ac.kr

**Keywords:** septic arthritis, native joint septic arthritis, bone and joint infections, *Staphylococcus aureus*, streptococci

## Abstract

**Background/Objectives**: This study aimed to evaluate and compare the clinical characteristics and outcomes of patients with methicillin-susceptible *Staphylococcus aureus* (MSSA) and streptococcal native joint septic arthritis (NJSA). **Methods**: This retrospective multicenter study included adult patients with NJSA from three tertiary care hospitals between 2004 and 2023. Patients were categorized into three groups based on bacterial virulence and differences among streptococcal species: MSSA, non-viridans streptococci, and viridans streptococci. **Results**: A total of 213 NJSA cases were identified, comprising 164 MSSA, 35 non-viridans streptococcal, and 14 viridans streptococcal cases. Non-viridans streptococcal NJSA exhibited significantly more acute clinical features associated with systemic inflammation, including frequent fever, leukocytosis, thrombocytopenia, elevated C-reactive protein levels, higher total bilirubin levels, and acute kidney injury, compared to MSSA and viridans streptococcal NJSA. The clinical presentations were similar between the MSSA and viridans streptococcal groups. The non-viridans streptococcal group showed a higher incidence of concomitant bacteremia, but a lower proportion of positive joint culture results. Treatment approaches were consistent across the groups, except for a shorter duration of antibiotic therapy in the viridans group. Treatment failure rates were comparable: 9.8% for MSSA, 14.3% for non-viridans streptococci, and 21.4% for viridans streptococci. **Conclusions**: This study highlights the heterogeneous nature of streptococcal NJSA, with non-viridans streptococci presenting more aggressive clinical manifestations despite similar treatment responses across groups. These findings challenge conventional views on streptococci as a less virulent pathogen than MSSA and emphasize the need for diagnostic and management strategies tailored to the unique pathogenic traits of these bacteria.

## 1. Introduction

Native joint septic arthritis (NJSA) is a debilitating condition caused by microbial invasion of synovial joints, with *Staphylococcus aureus*, particularly methicillin-susceptible *S. aureus* (MSSA), being the predominant pathogen worldwide [1]. *S. aureus* is recognized for its substantial virulence and strong association with invasive infections [2]. Although less common, streptococcal NJSA constitutes a significant proportion of cases and often presents as life-threatening complications [3]. Streptococci are categorized into viridans and non-viridans groups based on their pathogenic characteristics, which manifest in diverse clinical presentations [4]. Non-viridans species, such as *Streptococcus pyogenes* and *S. agalactiae* (GBS), are frequently implicated in severe systemic inflammation, whereas viridans streptococci tend to follow a more indolent course but may still cause complications, such as endocarditis. Despite these differences, there is limited research directly comparing the clinical characteristics and outcomes of streptococcal NJSA to those caused by *S. aureus* [5,6].

In this multicenter retrospective study, we aimed to evaluate the clinical characteristics and outcomes of NJSA caused by streptococci and *S. aureus* through direct comparison. Given that methicillin-resistant *S. aureus* (MRSA) differs from MSSA in terms of empirical antibiotic selection and potential virulence, MSSA was used to minimize variability in treatment outcomes [7,8]. Additionally, streptococci were divided into viridans and non-viridans groups to better understand their pathogenic differences and their impact on clinical outcomes.

## 2. Results

### 2.1. Microbiological Profile and Distribution of Causative Pathogens in NJSA

**Figure 1:**
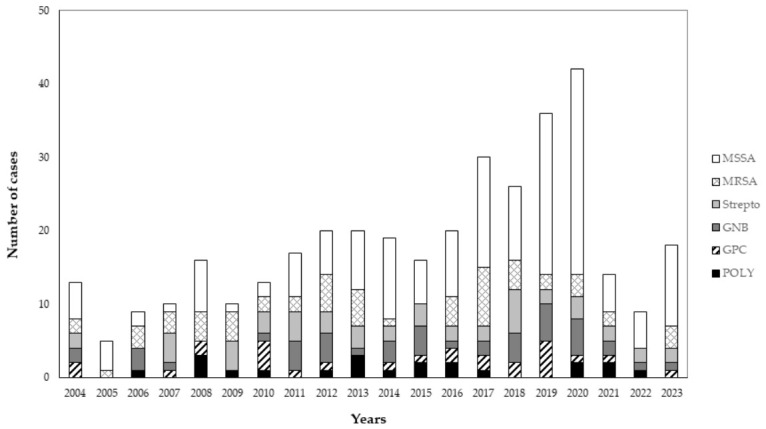
Proportion of native joint septic arthritis cases (2004–2023). MSSA, methicillin-susceptible *Staphylococcus aureus*; MRSA, methicillin-resistant *S. aureus*; Strepto, Streptococci; GNB, Gram-negative bacilli; GPC, Gram-positive cocci; POLY, polymicrobial infections.

During the study period, 363 patients with NJSA were identified. *S. aureus* was the most prevalent pathogen, accounting for 61% of cases, followed by *Streptococcus* (14%), Gram-negative bacilli (GNB, 12%), other Gram-positive cocci (7%), and polymicrobial infections (6%). MRSA comprised 16% of all cases and represented 26.1% of *S. aureus* isolates. The distribution of causative microorganisms remained consistent annually, with occasional alternations between *Streptococcus* and GNB in the second and third positions in specific years (Figure 1).

For comparison, 213 patients were analyzed, including 164 MSSA, 35 non-viridans streptococcal, and 14 viridans streptococcal infections. Non-viridans streptococci primarily included *S. agalactiae* (n = 15), *S. dysgalactiae* (n = 8), and *S. pyogenes* (n = 6) (Table 1). Viridans streptococcal NJSA cases were mainly caused by *S. mitis/oralis* (n = 10), with other species being less common. Microorganisms were identified in the joint fluid cultures of all patients, except for four MSSA, four non-viridans streptococcal, and two viridans streptococcal cases, where pathogens were isolated exclusively from blood samples. In one case, *S. suis*, classified as a viridans streptococcus, was isolated from both cerebrospinal fluid (CSF) and blood specimens. Echocardiography was performed within two days of bacteremia identification in all ten cases with blood-only isolates, and no vegetation was detected.

### 2.2. Demographic and Clinical Characteristics of S. aureus and Streptococcal NJSA

Table 2 compares the demographics, comorbidities, and clinical presentations of the groups. Comorbidities and joint distributions were generally similar, except for a higher frequency of hip joint involvement in the viridans streptococcal group. A history of intra-articular injection was more prominent in the MSSA group compared to the streptococcal groups, particularly the non-viridans streptococcal group (45.7% vs. 20%, *p* < 0.05).

The non-viridans streptococcal group exhibited more acute clinical features with systemic inflammation, including higher rates of fever (71.4% vs. 43.4% in MSSA), leukocytosis (median WBC: 16.4 vs. 11.6 × 10^3^/uL), thrombocytopenia (193 vs. 260 × 10^3^/uL), elevated C-reactive protein (CRP, median: 20.7 vs. 12.7 mg/dL), higher total bilirubin levels (median: 1.1 vs. 0.7 mg/dL), and acute kidney injury (37.5% vs. 12.0%) compared to the MSSA group. These clinical characteristics were largely similar between the non-viridans and viridans streptococcal groups. The MSSA and viridans streptococcal groups showed few differences, except for a higher prevalence of osteoarthritis in the viridans streptococcal group (42.9% vs. 14.6%, *p* < 0.05).

The non-viridans streptococcal group had a higher incidence of concomitant bacteremia (73.5% vs. 50.4% in MSSA and 25.0% in viridans streptococci), although they had a lower proportion of positive joint cultures than the other groups. Among the 103 patients who underwent echocardiography, 72 were in the MSSA group, 23 in the non-viridans streptococcal group, and 8 in the viridans streptococcal group. Infective endocarditis was diagnosed in only two MSSA cases and was not diagnosed in any patients with non-viridans or viridans streptococcal NJSA who underwent echocardiographic evaluation.

### 2.3. Treatment and Outcomes in MSSA and Streptococcal NJSA

Treatment approaches were similar across the groups. Most patients underwent initial joint drainage (90.8% with MSSA, 82.9% with non-viridans streptococci, and 85.7% with viridans streptococci), and appropriate antibiotics were promptly initiated in the majority of cases (Table 3). The only significant difference in treatment was the shorter duration of antibiotic therapy in the viridans streptococcal group (median: 44 vs. 56 days in non-viridans streptococci and 44 vs. 47 days in MSSA; both *p* < 0.05).

**Figure 2:**
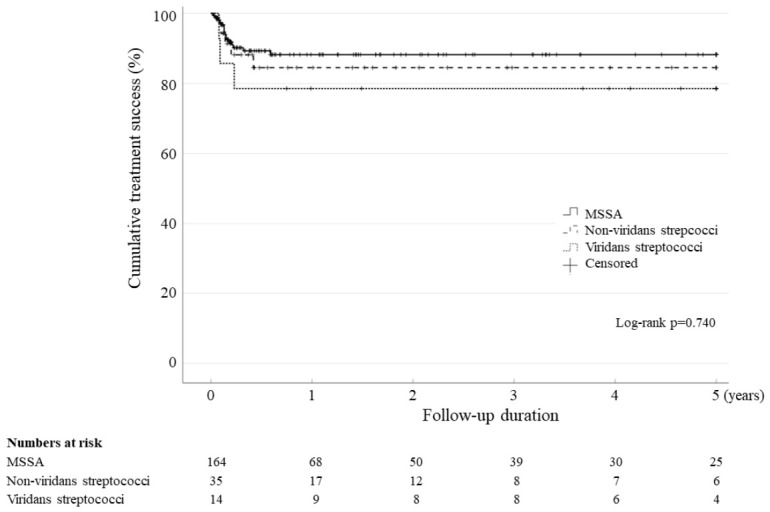
Kaplan–Meier analysis of treatment success in MSSA and streptococcal native joint septic arthritis (log-rank test, *p* = 0.740). MSSA, methicillin-susceptible *Staphylococcus aureus*.

The median follow-up duration was 8 months (interquartile range [IQR], 1–38 months; range, 0–186 months) in the time-to-event dataset. In total, 24 patients (11.3%) experienced treatment failure, including four deaths (1.9%), 12 cases (5.6%) requiring repeated surgical drainage after 30 days of antibiotic therapy, and 12 cases (5.6%) of relapse after completed therapy. Relapse occurred at a median of 28.5 days after discontinuation of antibiotic therapy (range, 9–582 days). Treatment failure rates showed slight variations: 9.8% for MSSA, 14.3% for non-viridans streptococci, and 21.4% for viridans streptococci. Mortality was rare, with one death in the viridans group and three in the MSSA group. Among cases requiring repeated surgical therapy after 30 days of antibiotic treatment, four relapsed after completing therapy: three with MSSA and one with *S. pyogenes*. Kaplan–Meier analysis demonstrated no statistically significant differences in treatment failure-free survival among the groups (Figure 2).

## 3. Discussion

NJSA remains a significant clinical concern due to its diverse etiologies and outcomes. This study provides a comprehensive analysis of NJSA caused by MSSA and streptococcal species, with a focus on the comparative profiles between non-viridans and viridans streptococci. The findings highlight the microbiological profile of streptococcal NJSA, particularly non-viridans streptococci, which are associated with more acute manifestations, systemic inflammation, and concomitant bacteremia than MSSA and viridans streptococcal NJSA. Despite this more aggressive presentation, treatment responses were similar across the groups.

This study confirms *Streptococcus* as the second most common pathogen in NJSA after MSSA, consistent with epidemiological trends [9,10]. Although *Streptococcus* is a well-recognized causative agent, the clinical characteristics of its subtypes remain insufficiently defined. Previous research has shown mixed results regarding the outcomes of streptococcal infections compared to *S. aureus*, particularly in prosthetic joint infections (PJIs) [11,12,13]. Some studies reported lower mortality rates for streptococcal septic arthritis, though they often included PJIs and lacked detailed laboratory data, limiting their relevance to NJSA [13,14]. One study discovered emerging Group B streptococcal (GBS) pathogens, a subset of non-viridans streptococci, as a favorable outcome predictor in NJSA, particularly in patients without underlying conditions [5]. While prior studies have described the variable clinical presentations and prognoses of streptococcal septic arthritis, they often generalized findings across *Streptococcus* species without exploring subtype-level characteristics [15,16,17,18].

Our results revealed that non-viridans streptococcal NJSA elicited a more robust inflammatory response than MSSA, characterized by higher rates of fever, leukocytosis, thrombocytopenia, and elevated CRP levels. Notably, the fever rate in non-viridans streptococcal infections exceeded the overall fever prevalence in NJSA, reported at approximately 60% [19]. The increased incidence of acute kidney injury (AKI) and elevated bilirubin levels further support the systemic nature of non-viridans streptococcal infections, possibly reflecting their capacity for toxin production and intense immune activation. In contrast, viridans streptococci exhibited a subacute and indolent clinical course, presenting similarly to MSSA. This may be attributed to their lower virulence. These differences between non-viridans and viridans streptococcal NJSA may be clinically relevant because grouping all streptococcal infections together could obscure subtype-specific considerations in presentation, diagnostic approach, and management. Therefore, non-viridans and viridans streptococci should not be regarded as a single homogeneous streptococcal group in NJSA. This interpretation emphasizes the multifaceted nature of streptococcal NJSA and is consistent with the greater virulence and systemic involvement of pathogens such as *S. pyogenes* and *S. agalactiae* [20,21]. In this context, the systemic toxic effects of non-viridans streptococcal infections, as well as those caused by *S. aureus*, should be recognized as contributors to severe NJSA cases [22].

In the present study, non-viridans streptococci were more frequently responsible for concomitant bacteremia but were less likely to yield positive joint cultures compared with MSSA and viridans streptococci. This pattern may suggest differences in joint tropism or pathogen dissemination pathways, with non-viridans streptococci exhibiting a higher propensity for hematogenous dissemination, which could explain the increased incidence of bacteremia [3]. Despite the frequent occurrence of bacteremia in non-viridans streptococcal NJSA, infective endocarditis was not diagnosed in this group. This finding contrasts with the higher rates of infective endocarditis observed in streptococcal vertebral osteomyelitis [23,24]. However, echocardiographic evaluation was performed in only approximately half of the study population because of the retrospective study design. Therefore, infective endocarditis may have been underdiagnosed, and the absence of diagnosed infective endocarditis should be interpreted with caution. Microbial virulence, host factors, and organism-specific behaviors may have contributed to the apparently low incidence of infective endocarditis in non-viridans streptococcal NJSA cases with bacteremia [25]. Further research is needed to clarify these mechanisms and pathophysiological processes and to explore the factors that determine the development of infective endocarditis in this setting.

The predominance of *S. agalactiae* and *S. pyogenes* among non-viridans cases aligns with their established roles in invasive infections and their strong connection to bacteremia in bone and joint infections, even in otherwise healthy individuals [16,26,27]. Although the non-viridans streptococcal group is heterogeneous, shared virulence factors among these pathogens may enhance their ability to invade the bloodstream and disseminate. This reinforces the hypothesis that hematogenous spread is the primary route of bone and joint infection [28,29]. Despite the higher rates of bacteremia, the lower culture positivity rates of non-viridans streptococci in joint fluid may hinder pathogen isolation, possibly due to biofilm formation or rapid systemic dissemination. These findings stress the importance of obtaining blood cultures and incorporating adjunctive molecular diagnostic methods to improve pathogen detection in synovial fluid for the management of septic arthritis. Such approaches not only guide targeted antimicrobial therapy but also facilitate the prompt detection of bacteremia, enhancing timely intervention and alerting clinicians to potential complications.

Despite variations in clinical severity, similar treatment failure rates across groups indicate that early and appropriate management is crucial for favorable outcomes, regardless of the causative organism [30]. In our study, treatment strategies, including joint drainage and antibiotic therapy, were consistent across groups. However, differences in antibiotic duration were observed among the groups, with the viridans streptococcal group receiving the shortest course, followed by the MSSA group, and the non-viridans streptococcal group receiving the longest course. These findings raise the possibility that treatment duration may vary by causative pathogen and could be individualized according to patient- and pathogen-related factors. Nevertheless, these data limit our ability to determine pathogen-specific differences in optimal antibiotic duration because of the small sample sizes of the streptococcal subgroups and the retrospective study design. Prospective, adequately powered studies are warranted to validate these findings and inform the implementation of antibiotic protocols tailored to clinical severity, treatment response, source control, and pathogen group.

This study had some limitations. First, its retrospective design and reliance on data from tertiary care hospitals may restrict generalizability, with a potential selection bias towards more serious cases. Variations in treatment protocols across centers could also alter the prognosis. Although the exclusion criteria were applied to ensure that the study population reflected isolated bacterial native joint septic arthritis, the exclusion of cases with suspected or confirmed osteomyelitis may have affected the assessment of disease severity, particularly in cases with adjacent bone involvement. Second, missing data and the small sample sizes of the streptococcal groups, particularly the viridans streptococcal group, due to the rarity of NJSA, further limit its applicability [1]. This imbalance across pathogen groups may have reduced the statistical power to detect significant differences in treatment failure and may have increased the possibility of a Type II error. Given the small number of treatment failure events, additional multivariable analyses to identify independent factors associated with severe presentation or treatment failure were not feasible. Third, available clinical records did not allow assessment of functional outcomes or long-term joint-related complications, such as joint dysfunction or sequelae, which could have provided additional prognostic insights. Lastly, the nearly two-decade study period may introduce temporal bias, as evolving diagnostic techniques and treatment strategies likely influenced the results.

## 4. Materials and Methods

### 4.1. Study Population and Design

In this retrospective study, we examined adult patients diagnosed with NJSA at three tertiary-care university hospitals in South Korea between 2004 and 2023. Eligible participants were aged ≥18 years, had bacterial strains isolated from joint fluid and/or blood, and were followed up for at least 1 month after completing treatment. Exclusion criteria included cases with unidentified microorganisms, polymicrobial infections, non-bacterial pathogens, prosthetic joint materials, suspected or confirmed osteomyelitis (e.g., diabetic foot ulcers), and insufficient post-treatment follow-up. These exclusion criteria were applied to focus the analysis on isolated bacterial native joint septic arthritis and to minimize confounding from conditions associated with distinct treatment strategies and clinical outcomes. From the initial pool of NJSA cases, those caused by *S. aureus* and streptococci were selected and categorized into three groups: MSSA, non-viridans streptococci, and viridans streptococci.

Septic arthritis was defined as meeting at least one of the following criteria: isolation of a pathogenic organism from the affected joint, isolation of a pathogenic organism from another source (e.g., blood) with clinical evidence of a swollen joint, typical clinical features of septic arthritis, such as turbid joint fluid, during antibiotic treatment, or pathological findings suggestive of septic arthritis [19].

### 4.2. Data Collection

Data were collected on patient demographics, comorbidities, clinical presentations, laboratory findings at admission, identified pathogens, and medical and surgical management. Appropriate antibiotic therapy was defined as administration of an antimicrobial agent active against the identified pathogen. Early appropriate antibiotic therapy was defined as initiation within 48 h of diagnosis. The total duration of antibiotic therapy was calculated from the date of initiation of appropriate antibiotic therapy to the date of discontinuation. Intravenous and oral antibiotic durations were recorded separately. Drainage methods included repeated arthrocentesis, arthroscopic debridement, or open arthrotomy. For cases requiring surgery to address persistent infection during antibiotic therapy, the number of surgical debridements was recorded. Surgical procedures such as arthroplasty, arthrodesis, or amputation to improve joint function were excluded from the debridement count. The choice of drainage procedure was determined at the discretion of the treating physicians based on the clinical condition of each patient. As the causative microorganisms are major pathogens in infective endocarditis, echocardiograms performed within 2 weeks of diagnosis were also noted. Two clinicians at each hospital meticulously reviewed medical records, with any discrepancies or conflicting data resolved through cross-verification by two authors.

### 4.3. Outcome

Outcomes were classified as treatment success or failure. Treatment success was defined as the resolution of infection-related signs and symptoms at the end of therapy, regardless of residual joint dysfunction or sequelae. Treatment failure was subdivided into three categories: (i) “Death,” referring to all-cause mortality within 30 days of diagnosis; (ii) “Repeated surgical drainage after 30 days of antibiotic therapy,” representing the need for additional surgical drainage due to persistent infection despite 30 days of antibiotic treatment, and (iii) “Relapse after completed therapy,” signifying the recurrence of symptoms in previously affected joints after completing antibiotic therapy, attributed to reinfection or persistent microorganisms. Subcategory (ii) was confirmed by clinical signs, symptoms, and elevated acute-phase reactants, such as C-reactive protein (CRP) or erythrocyte sedimentation rate (ESR), after initial improvement or by re-isolating the same microorganism from new samples during treatment.

### 4.4. Statistical Analysis

Statistical analyses were performed using SPSS version 29.0 (IBM Corp., Armonk, NY, USA). A *p*-value of <0.05 (two-tailed) was considered statistically significant. Continuous variables were analyzed using Student’s *t*-test or the Mann–Whitney test, while categorical variables were compared using the χ^2^ test or Fisher’s exact test. The Kaplan–Meier method was used to calculate cumulative treatment failure-free survival rates. For patients with overlapping treatment failure categories, each patient was counted once in the overall treatment failure analysis, and the earliest event date was used for the time-to-event analysis.

## 5. Conclusions

Our study bridges this gap by directly comparing the two streptococcal subtypes with MSSA, offering unique data on laboratory results, management approaches, and prognosis. Non-viridans streptococci present with more severe clinical manifestations than MSSA and viridans streptococci, though treatment responses are comparable. These findings challenge conventional perspectives on streptococcal infections and underscore the need for pathogen-specific approaches to diagnosis and treatment. Future research should prioritize prospective studies and molecular characterization to elucidate the pathophysiology and optimize therapeutic strategies for NJSA.

## Figures and Tables

**Table 1:** Clinical characteristics of patients with MSSA and streptococcal native joint septic arthritis.

Variables	MSSA (n = 164)	*Streptococcus*		
		Non-Viridans (n = 35)	Viridans (n = 14)	All (n = 49)
Age, years	67 (56–74)	64 (57–73)	69 (59–79)	65 (57–73)
Male	82 (50.0)	18 (51.4)	6 (42.9)	24 (49.0)
Comobidities				
Immunocompromised status	21 (12.8)	3 (8.6)	1 (7.1)	4 (8.2)
Solid tumor	8 (4.9)	1 (2.9)	1 (7.1)	2 (4.1)
Hematologic malignancy	2 (1.2)	1 (2.9)	0 (0)	1 (2.0)
Immunosuppressant agents	15 (9.1)	2 (5.7)	0 (0)	2 (4.1)
Hemodialysis	3 (1.8)	3 (8.6)	1 (7.1)	4 (8.2)
Diabetes mellitus	53 (32.3)	12 (34.3)	4 (28.6)	16 (32.7)
Rheumatoid arthritis	6 (3.7)	0 (0)	0 (0)	0 (0)
Osteoarthritis ^b^	24 (14.6)	6 (17.1)	6 (42.9)	12 (24.5)
Charlson comorbidity score	3 (2–4)	3 (2–4)	4 (1.8–5)	3 (2–4.5)
Previous joint intervention				
Intra-articular injection ^a,d^	75 (45.7)	7 (20.0)	4 (28.6)	11 (22.4)
Acupuncture	12 (7.3)	1 (2.9)	1 (7.1)	2 (4.1)
Arthroscopic procedure	5 (3.0)	0 (0)	0 (0)	0 (0)
Involved joints				
Knee	69 (42.1)	19 (54.3)	8 (57.1)	27 (55.1)
Hip ^b,d^	15 (9.1)	6 (17.1)	4 (28.6)	10 (20.4)
Shoulder ^d^	46 (28.0)	4 (11.4)	1 (7.1)	5 (10.2)
Elbow	12 (7.3)	2 (5.7)	0 (0)	2 (4.1)
Wrist	5 (3.0)	1 (2.9)	0 (0)	1 (2.0)
Ankle	9 (5.5)	2 (5.7)	1 (7.1)	3 (6.1)
Small joints	12 (7.3)	3 (8.6)	0 (0)	3 (6.1)
Polyarthropathy	7 (4.3)	3 (8.6)	0 (0)	3 (6.1)
Combined periarticular abscess	41 (25.0)	11 (31.4)	3 (21.4)	14 (28.6)
Combined pyogenic spondylitis	5 (3.0)	3 (8.6)	0 (0)	3 (6.1)
Endocarditis	2 (2.8)	0 (0)	0 (0)	0 (0)
Clinical presentation				
Fever on admission ≥ 38 °C ^a,d^	69 (43.4)	25 (71.4)	7 (50.0)	32 (65.3)
Shock	6 (3.7)	2 (5.7)	1 (7.1)	3 (6.1)
Acute kidney injury ^a,b,d^	19 (12.0)	12 (37.5)	5 (38.5)	12 (27.3)
Laboratory test at admission				
WBC, ×10^3^/uL ^a,c^	11.6 (8.9–14.2)	16.4 (11.1–24)	10.7 (8.8–11.8)	12.9 (10.7–20.3)
Hemoglobin (g/dL)	12.1 (10.9–13.4)	12.3 (10.9–13.6)	11.5 (9.7–13.2)	12.3 (10.7–13.5)
Platelet, ×10^3^/uL ^a^	260 (190–334)	193 (147–246)	233 (183–366)	215 (154–253)
CRP (mg/dL) ^a,c^	12.7 (6.2–21.0)	20.7 (14.5–28.3)	11.5 (5.1–18.5)	17.9 (11.2–26.7)
ESR (mm/h)	68 (46–99)	59 (41–100)	89 (24–103)	78 (37–101)
Total bilirubin (mg/dL) ^a^	0.7 (0.5–1.0)	1.1 (0.6–1.8)	0.7 (0.4–1.1)	0.9 (0.6–1.5)
Serum glucose > 180 mg/dL	46 (29.5)	8 (25.8)	3 (23.1)	11 (25.0)
Creatinine > 1.6 mg/dL ^a,d^	16 (10.0)	12 (34.3)	3 (21.4)	15 (30.6)
Serum sodium (mEq/L) ^a,b,d^	137 (134–140)	134 (131–137)	138 (135–141)	135 (132–138)
Synovial fluid WBC, ×10^3^/mm^3^	91 (42–168)	104 (55–179)	64 (34–139)	96 (47–175)
Positive joint culture ^a,d^	156 (97.5)	27 (81.8)	12 (92.3)	39 (84.8)
Concomitant bacteremia ^a,c^	59 (50.4)	25 (73.5)	3 (25.0)	28 (60.9)

Data were expressed as numbers (%) unless otherwise indicated. Continuous variables were expressed as the median and interquartile range (IQR). The proportion was calculated based on 103 cases of echocardiography, 206 cases of joint culture, and 163 cases of blood culture. Among 206 patients with available joint culture results, 11 had negative joint cultures; 10 had joint specimens obtained before antibiotic administration, and one patient with sacroiliac joint involvement underwent operative joint culture after 4 days of antibiotic therapy following blood culture-based diagnosis. The proportion of acute kidney injury cases was analyzed, excluding those involving hemodialysis. NJSA, native joint septic arthritis; MSSA, methicillin-susceptible *Staphylococcus aureus*. ^a^ *p* < 0.05, between MSSA NJSA and non-viridans NJSA. ^b^ *p* < 0.05 between MSSA NJSA and viridans NJSA. ^c^ *p* < 0.05 between non-viridans NJSA and viridans NJSA. ^d^ *p* < 0.05 between MSSA and Streptococcal NJSA.

**Table 2:** Comparison of treatment and outcomes between MSSA and streptococcal native joint septic arthritis.

Variables	MSSA (n = 164)	*Streptococcus*		
		Non-Viridans (n = 35)	Viridans (n = 14)	All (n = 49)
Initial drainage modes	149 (90.8)	29 (82.9)	12 (85.7)	41 (83.7)
Time to drainage, days	1 (0–3)	1 (0–4)	2 (1–5)	1 (0–4)
Drainage ≤ 48 h	105 (70.0)	19 (65.5)	8 (66.7)	27 (65.9)
Repeated arthrocentesis	12 (7.3)	3 (8.6)	1 (7.1)	4 (8.2)
Arthroscopy	93 (56.7)	18 (51.4)	9 (64.3)	27 (55.1)
Arthrotomy	44 (26.8)	8 (22.9)	2 (14.3)	10 (20.4)
Surgical drainage ≥ 2 times	29 (17.7)	6 (17.1)	3 (21.4)	9 (18.3)
Appropriate antibiotics ≤ 48 h	151 (92.1)	30 (85.7)	12 (85.7)	42 (85.7)
Duration of antibiotic therapy, days ^b,c^	47 (33–70)	56 (37–74)	44 (38–62)	51 (38–69)
Intravenous antibiotics ^a,d^	24 (16–38)	31 (21–43)	29 (26–42)	29 (22–43)
Oral antibiotics ^b,c^	19 (8–36)	25 (6–37)	13 (0–21)	15 (3–33)
Treatment failure	16 (9.8)	5 (14.3)	3 (21.4)	8 (16.3)
Death	3 (1.8)	0 (0)	1 (7.1)	1 (2.0)
Repeated surgical drainage after 30 days of antibiotic therapy	7 (4.3)	3 (8.6)	2 (14.3)	5 (10.2)
Relapse after completed therapy	9 (5.5)	3 (8.6)	0 (0)	3 (6.1)

Data were expressed as numbers (%) unless otherwise indicated. Continuous variables were expressed as median and interquartile range (IQR). Surgical drainage includes arthrotomy, arthroscopic incision, and drainage. NJSA, native joint septic arthritis; MSSA, methicillin-susceptible *Staphylococcus aureus*. ^a^ *p* < 0.05, between MSSA NJSA and non-*viridans* NJSA. ^b^ *p* < 0.05 between MSSA NJSA and viridans NJSA. ^c^ *p* < 0.05 between MSSA NJSA and *viridans* NJSA. ^d^ *p* < 0.05 between MSSA and Streptococcal NJSA.

**Table 3:** Causative microorganisms in 49 cases of streptococcal native joint septic arthritis.

*Streptococcus* Species		Cases (n)
Non-viridans streptococci	*S. agalactiae*	15
	*S. dysgalactiae*	8
	*S. pyogenes*	6
	*S. pneumoniae*	5
	*S. gallolyticus*	1
Viridans streptococci	*S. mitis/oralis*	10
	*S. salivarius*	1
	*S. anginosus*	1
	*S. sanguis*	1
	*S. suis*	1

## Data Availability

The data supporting the conclusions of this article will be made available by the authors upon request. The datasets are not publicly available due to privacy and ethical restrictions.

## Abbreviations

AKI	acute kidney injury
CRP	C-reactive protein
CSF	cerebrospinal fluid
ESR	erythrocyte sedimentation rate
GNB	Gram-negative bacilli
MRSA	methicillin-resistant *S. aureus*
MSSA	methicillin-susceptible *Staphylococcus aureus*
NJSA	native joint septic arthritis
